# Quantifying cell fate change under different stochastic gene activation frameworks

**DOI:** 10.1002/qub2.82

**Published:** 2024-11-21

**Authors:** Xinxin Chen, Ying Sheng, Liang Chen, Moxun Tang, Feng Jiao

**Affiliations:** ^1^ Guangzhou Center for Applied Mathematics Guangzhou University Guangzhou China; ^2^ Department of Mathematics Michigan State University East Lansing Michigan USA

**Keywords:** cell fate change, mRNA distribution, statistical transcription threshold, stochastic gene transcription, transcription noise

## Abstract

Gene transcription is a stochastic process characterized by fluctuations in mRNA levels of the same gene in isogenic cell populations. A central question in single‐cell studies is how to map transcriptional variability to phenotypic differences between isogenic cells. We introduced a measurable and statistical transcription threshold *I* for critical genes that determine the entry level of Waddington’s canal toward a specific cell fate. Subsequently, *J*_*I*_, which is the probability that a cell has at least *I* mRNA molecules of a given gene, approximates the likelihood of a cell committing to the corresponding fate. In this study, we extended the previous results of *J*_*I*_ of the classical telegraph model by considering more complex models with different gene activation frameworks. We showed that (a) the upregulation of the critical gene may significantly suppress cell fate change and (b) increasing transcription noise performs a bidirectional role that can either enhance or suppress the cell fate change. These observations matched accurately with the data from bacterial, yeast, and mammalian cells. We estimated the threshold *I* from these data and predicted that (a) the traditional human immunodeficiency virus (HIV) activators that modulate gene activation frequency at high doses may largely suppress HIV reactivation and (b) the cells may favor noisier (or less noisy) regulation of stress genes under high (or low) environmental pressures to maintain cell viability.

## 1 INTRODUCTION

Gene transcription is a stochastic process, manifested by fluctuations in mRNA levels of the same gene in an isogenic cell population [1, 2, 3]. The mRNA copy number distribution data provides a conventional quantification of fluctuations [4, 5, 6]. This sets a statistical basis for approximating the mass function *P*_*m*_ (probability of having exactly *m* copies of mRNA molecules in one cell) and the transcription noise *CV* ^2^ (the variance of mRNA copy numbers over the mean squared) [4, 6, 7]. The mass function *P*_*m*_ depicts a panoramic view of transcriptional stochasticity whereas the noise at steady state is a single number that quantifies the deviation of mRNA numbers in individual cells from the mean *E*. When combined, these concepts may signify a phenotypic trend in the cell population [8, 9, 10].

A central question in single‐cell studies is how to map the transcription variability to phenotypic differences between cells within a genetically identical population [11, 12, 13]. When a cell differentiates, its fate is usually determined by the transcriptional state of one or more critical genes [14, 15]. For example, retinoblastoma gene *RB1* plays a key role in determining the fate of osteoblasts and adipocytes in mesenchymal stem cells as silencing *RB1* impairs bone differentiation while expanding the fat compartments in mouse [16]. The transcriptional status of *sox10* is associated with tumor formation, as seen in the melanocytes of zebrafish, in which forced overexpression of *sox10* accelerated melanoma initiation whereas inactivation of *sox10* delayed the onset of melanoma [17].

**FIGURE 1:**
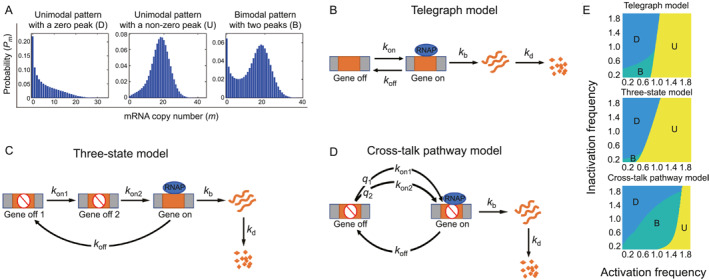
mRNA distribution and stochastic transcription models. (A) mRNA distribution *P*_*m*_ that quantifies the probability of *m* mRNA molecules of the interested gene in an isogenic cell. The following distribution patterns are commonly observed: the unimodel pattern for which *P*_*m*_ has a unique zero or nonzero peak (left or middle panels) and the bimodal pattern for which *P*_*m*_ has two peaks (right panel). (B) The classical telegraph model in which the gene switches randomly between *off* and *on* states. mRNAs are synthesized only when the gene is *on*. Each process is controlled by a single rate‐limiting step with constant rate. (C) The three‐state model in which the gene is activated through two sequential rate‐limiting steps. (D) The cross‐talk pathway model in which the gene is competitively activated through two parallel rate‐limiting steps. (E) Phase diagrams of the three distribution shapes shown in (A) for the telegraph, three‐state, and cross‐talk pathway models. Here *k*_on_ ∈ [0.1, 2] for the telegraph model, *k*_on1_ = *k*_on2_ ∈ [0.2, 4] for the three‐state model, *k*_on1_ = 0.1, *k*_on2_ = 2, *q*_2_ ∈ (0, 1) for the cross‐talk pathway model, and *k*_b_ = 5, *k*_off_ ∈ [0.1, 2] for all models.

Many studies have focused on how mRNA distribution patterns of the critical gene are associated with cell phenotypic difference [2, 9, 18]. The unimodel distribution (*P*_*m*_ has a unique zero or nonzero peak) suggests phenotypic homogeneity of an isogenic cell population while the bimodal distribution (*P*_*m*_ has two peaks) supports two sub‐populations of isogenic cells with distinct cell fates; see Figure 1A. For instance, the pluripotency of mouse embryonic stem cells is associated with two mRNA distribution peaks of the *Nanog* gene. Low expression (first distribution peak) of *Nanog* in stem cells is essential for retaining stemness whereas cells with high expression (second distribution peak) of *Nanog* are more likely to differentiate [19]. In the discussion of connecting bimodal mRNA distribution with cell fate, there is an implicit assumption of transcription threshold (denoted by *I*) between two distribution peaks, such that the cell is stabilized within the current state when the critical gene is silent or expressed below *I* or the cell changes its fate when the critical gene is expressed over *I* [13, 20].

The transcription threshold *I* is also qualified in Conrad Waddington’s “epigenetic landscape”, where these critical genes function as binary switches that drive the cells to proceed along one branching canal or another that restricts the cell fate [15, 21, 22]. When the mRNA copy number of critical genes exceeds the altitude of the canal entrance, the threshold value *I* in Waddington’s landscape, the cell is likely to jump into the canal [14, 15]. For example, *Sox17* is a potent inducer of stem cell in mice toward the extraembryonic endoderm state, requiring only the overexpression of *Sox17* to certain levels to initiate the related regulatory networks [10, 23].

However, a deterministic transcription threshold *I* may not exist. For instance, the p53 transcription factor (TF) must reach a threshold level to induce cell apoptosis in response to DNA damage whereas both surviving and dying colon cancer cells under chemotherapy reach similar levels of p53, indicating that there is no fixed p53 threshold *I* that determines cell death [24, 25]. This observation suggests that there are additional factors such as apoptosis inhibitors that can contribute to changes in cell fate; thus, p53 may play a critical but partial role in determining cell death. Therefore, the threshold *I* of the critical gene should be quantified statistically because a number of other hidden factors influence cell fate changes [24, 26].

In a recent study [27], a statistical threshold *I* has been defined to demonstrate how transcription noise influences changes in the cell fate. For an isogenic cell population in a stable environment, let *R* be the percentage of cells toward a certain fate and *J*_*i*_ be the distribution

(1) $$\begin{array}{c} J_{i}=\sum_{m=i}^{\infty }P_{m} \end{array}$$

for each *i* = 0, 1, ⋯. Here we call *J*_*i*_ the jumping index, which quantifies the probability that a cell has at least *i* mRNA molecules for a critical gene. As *R* ∈ (0, 1] and *J*_*i*_ decrease with *i* from 1 to 0, there must be a unique integer *I* ≥ 1 such that *R* ∈ (*J*_*I*_, *J*_*I*‒1_]. We denote the transcription threshold by *I*, which quantifies that when a cell has at least *I* transcripts of the critical gene, it has a probability of *J*_*I*_ (or approximately *R*) to commit the corresponding fate. The threshold *I* can be estimated using the cell fate change data *R* and mRNA distribution data *P*_*m*_ and supports the observation that cell fate changes may occur even if the transcript number is less than *I* or the cell maintains the current fate with more than *I* transcripts [24].

In this study, we used *J*_*I*_ to approximate the rate of change in cell fate in an isogenic cell population. We explored how the mean transcription level and noise, combined with different gene activation frameworks in mathematical models, directly affect cell fate. One of the most prevalent mathematical models that characterize stochastic gene transcription in single cells is the telegraph model [2, 20, 28], as depicted in Figure 1B. In this model, a gene randomly switches between the *off* and *on* states, with an activation rate of *k*_on_ > 0 and inactivation rate of *k*_off_ > 0. The mRNA molecules are produced only when the gene is active, with a birth rate of *k*_b_ > 0 and degradation rate of *k*_d_ > 0.

However, the telegraph model cannot capture all genetic regulations, such as the gene activation process modulated by the multistep recruitment of TFs or competition of multiple pathways [1, 29]. Integrating detailed mechanisms into the telegraph model generates relatively complex models [30, 31, 32, 33]; see Figure 1C,D. In this study, we analyzed the modulation of *J*_*i*_ using different stochastic gene transcription models. We extend the previous results of *J*_*i*_ for a simple telegraph model and show that an increase in the transcription mean or noise can suppress or enhance *J*_*i*_ under different cellular conditions. These observations can be further utilized to illustrate strategies for reactivation of human immunodeficiency virus (HIV) from its latency and the circumstances under which transcription noise can be beneficial for cells under environmental pressures.

## 2 RESULTS

### 2.1 Exact formulas of the jumping index *J*_*i*_

Let *N* be a random variable that counts the number of mRNA molecules of a target gene in a single cell of an isogenic population. The mass function *P*_*m*_ = Prob{*N* = *m*} is the probability that there are exactly *m* molecules in the cell, *m* = 0, 1, 2, ⋯, and the jumping index *J*_*i*_ defined in Equation (1), or called the complementary cumulative distribution function, is the probability that there are at least *i* molecules in the cell. *P*_*m*_, *J*_*i*_, and the mean value *E* are related by the following equation:

(2) $$\begin{array}{c} E=\sum_{m=0}^{\infty }mP_{m}=\sum_{i=1}^{\infty }J_{i}, \end{array}$$

where the first equality follows from the definition of *E* and the second equality follows from the identity in Ref. [34] (Page 56). By definition, $J_{0}=\sum_{m=0}^{\infty }P_{m}=1$. To determine *J*_*i*_ for *i* ≥ 1, we turn to finding *P*_*m*_ first. For this purpose, we consider the probability generating function of *N* defined as the expectation of *z* ^*N*^:

$$F\left(z\right)=E\left(z^{N}\right)=\sum_{m=0}^{\infty }z^{N}\text{Prob}\left\{N=m\right\}=\sum_{m=0}^{\infty }z^{N}P_{m}.$$

We use gene transcription models to derive differential equations of F(z) (Supplementary). By solving these equations, we obtain an exact formula of F(z), and then find $P_{m}$ by the reversion

(3) $$\begin{array}{c} P_{m}=\frac{1}{m!}\frac{\partial^{m}F\left(z\right)}{z^{m}}{|}_{z=0} \end{array}.$$

For the telegraph model illustrated in Figure 1B, the generating function *F*(*z*) is given by the confluence hypergeometric function _1_ *F*_1_ [28] (see Supplementary for its derivation):

F(z)=F11kon;kon+koff;kb(z−1)

Here, we set *k*_d_ = 1 for the sake of convenience [28, 35]. This is not an arbitrary choice but stems from the fact that the time and parameters can always be re‐normalized by the decay rate *k*_d_. Specifically, the time should be understood to be nondimensional and equal to the real time multiplied by *k*_d_, while the *k*_b_, *k*_on_ and *k*_off_ should also be understood to be nondimensional and equal to their real values divided by *k*_d_. The exact form of *P*_*m*_ for the telegraph model can be obtained using Equation (3) [28]:

Pm=kbmm!Γkon+koffΓkon+mΓkonΓkon+koff+mF11kon+m,kon+koff+m,−kb,

where Γ(·) is gamma function.

We also plot a phase diagram of three distribution shapes in the plane of the inactivation frequency *k*_off_ against the activation frequency *k*_on_; see Figure 1E. It shows that the bimodal distribution region satisfies *k*_on_ < 1 and *k*_off_ < 1, and the increase of *k*_on_ or *k*_off_ transits the bimodality to the unimodality [18]. The mean level *E* and noise *CV* ^2^ can be calculated by the system of ordinary equations or can be recovered by taking the derivatives of *F*(*z*) at *z* = 1 [28]:

$$E=\frac{dF\left(z\right)}{dz}{|}_{z=1},{\;CV}^{2}=\frac{\frac{d^{2}F\left(z\right)}{dz^{2}}{|}_{z=1}+E-E^{2}}{E^{2}}$$

This gives [28, 36]:

(4) $$\begin{array}{c} E=\frac{{k_{b}k}_{\text{on}}}{k_{\text{on}}+k_{\text{off}}},{\;CV}^{2}=\frac{\left(k_{\text{on}}+k_{\text{off}}\right)\left(k_{\text{on}}+1\right)}{k_{\text{on}}\left(k_{\text{on}}+k_{\text{off}}+1\right)}+\frac{1}{E}-1, \end{array}$$

for the telegraph model.

The three‐state model illustrated in Figure 1C, is supported by experimentally observing a universal exponentially distributed gene *on* duration [1, 37] and a unique peak in the gene *off* duration distribution for mouse fibroblast genes [37] and *Escherichia coli* (*E.coli*) *tetA* promoter [38]. The unimodality of *off* duration distribution can be best described by at least two ordered rate‐limiting steps that direct gene activation [33, 37, 38]. The behavior of the three‐state model is determined by two sequential activation steps with rates *k*_on1_ and *k*_on2_, the inactivation rate *k*_off_, and the synthesis rate *k*_b_. For the three‐state model, the steady‐state analytical formulas for *P*_*m*_ are expressed in the form of all system parameters over the degradation rate *k*_d_ [33, 39]. For convenience, we set *k*_d_ = 1 in what follows.

Subsequently, the corresponding generating function F(z) is given by the generalized hypergeometric function F22 [33, 39] (see Supplementary for its derivation):

F(z)=F22kon1,kon2;α,β;kb(z−1)

where *α* + *β* = *k*_on1_ + *k*_on2_ + *k*_off_ and *αβ* = *k*_on1_ *k*_on2_ + (*k*_on1_ + *k*_on2_)*k*_off_. Then the exact form of *P*_*m*_ for the three‐state model can be obtained using Equation (3) [33]:

Pm=kbmm!Γ(α)Γ(β)Γkon1+mΓkon2+mΓkon1Γkon2Γ(α+m)Γ(β+m)F22kon1+m,kon2+m;α+m,β+m;−kb.

We show a phase diagram of the three distribution shapes in the plane of the inactivation frequency *k*_off_ against the activation frequency *k*_on1_ *k*_on2_/(*k*_on1_ + *k*_on2_); see Figure 1E. It shows a smaller bimodal distribution region when compared with that of the telegraph model, confirming the conclusion that increasing the number of gene off states tends to suppress bimodal distribution under the same transcription mean level [40]. By solving the corresponding system of ordinary equations or by taking derivatives of *F*(*z*) at *z* = 1, we further obtained the mean *E* and noise *CV* ^2^ [33, 41]:

(5) $$E=\frac{{k_{b}k_{\text{on}1}k}_{\text{on}2}}{k_{\text{on}1}k_{\text{on}2}+\left(k_{\text{on}1}+k_{\text{on}2}\right)k_{\text{off}}},{\;CV}^{2}=\frac{\alpha \beta }{k_{\text{on}1}k_{\text{on}2}}\frac{\left(k_{\text{on}1}+1\right)\left(k_{\text{on}2}+1\right)}{\left(1+\alpha \right)\left(1+\beta \right)}+\frac{1}{E}-1,$$

for the three‐state model.

The cross‐talk pathway model, shown in Figure 1D, illustrates the competitive promoter binding between two types of TFs. One type of TF is activated by a weak basal pathway with rate *k*_on1_ and the other is activated by a strong signaling pathway with rate *k*_on2_ > *k*_on1_ [36, 42, 43]. The competition between two parallel pathways is signified by selection probabilities of *q*_1_ and *q*_2_, which satisfy *q*_1_ + *q*_2_ = 1 for each pathway. The generating function *F*(*z*) of the cross‐talk pathway model is given by the authors of Ref. [44] (see Supplementary for its derivation):

F(z)=F22kon1,kon2;α‾,β‾;kb(z−1)

with $\bar{\alpha }+\bar{\beta }=k_{\text{on}1}+k_{\text{on}2}+k_{\text{off}}$ and $\bar{\alpha }\bar{\beta }=k_{\text{on}1}k_{\text{on}2}+\left(q_{2}k_{\text{on}1}+q_{1}k_{\text{on}2}\right)k_{\text{off}}$. Here $k_{\text{on}1},k_{\text{on}2},k_{\text{off}}$ and $k_{b}$ should be understood to be nondimensional and equal to their real values divided by $k_{d}$. The exact form of $P_{m}$ for the cross‐talk pathway model can be obtained using Equation (3) [44]:

Pm=kbmm!Γα‾Γβ‾Γkon1+mΓkon2+mΓkon1Γkon2Γα‾+mΓβ‾+mF22kon1+m,kon2+m;α‾+m,β‾+m;−kb.

We show a phase diagram of the three distribution shapes in the plane of the inactivation frequency *k*_off_ against the activation frequency *k*_on1_ *k*_on2_/*k*_on1_ *k*_on2_/(*q*_2_ *k*_on1_ + *q*_1_ *k*_on2_); see Figure 1E. It displays a much larger bimodal distribution region compared with that of the telegraph and three‐state models, confirming the conclusion that the cross‐talk pathway model is more likely to generate the bimodal distribution under the same transcription mean level [40, 44]. By solving the corresponding system of ordinary equations or by taking the derivatives of *F*(*z*) at *z* = 1, we obtained the mean *E* and noise *CV* ^2^ [36, 45]:

(6) $$E=\frac{k_{b}}{1+\left(q_{1}/k_{\text{on}1}+q_{2}/k_{\text{on}2}\right)k_{\text{off}}},{\;CV}^{2}=\frac{\bar{\alpha }\bar{\beta }}{k_{\text{on}1}k_{\text{on}2}}\frac{\left(k_{\text{on}1}+1\right)\left(k_{\text{on}2}+1\right)}{\left(1+\bar{\alpha }\right)\left(1+\bar{\beta }\right)}+\frac{1}{E}-1,$$

for the cross‐talk pathway models.

### 2.2 Upregulation of the critical gene may suppress the cell fate change

**FIGURE 2:**
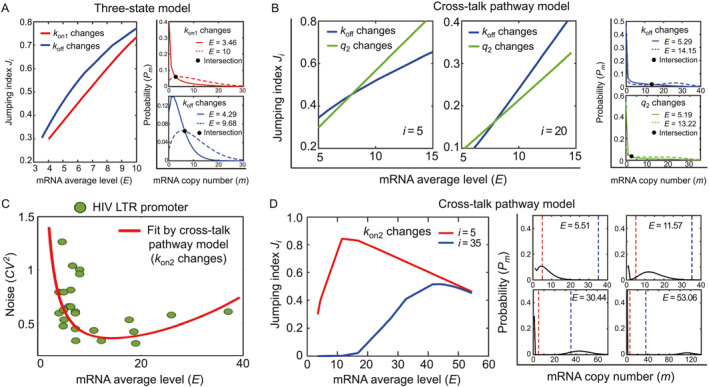
Correlation between the average mRNA level *E* and jumping index *J*_*i*_. The average level *E* is modulated by changing a single system parameter. (A) For the three‐state model, *J*_*i*_ is always positively correlated with *E* (left panel). Each pair of mRNA distributions under different *E* intersect only once (right panels). (B) For the cross‐talk pathway model, *J*_*i*_ is positively correlated with *E* if the gene inactivation rate or pathway selection probability is modulated (left panel). Each pair of mRNA distributions under different *E* intersect only once (right panels). (C) The transcription data (green circles) of noise *CV* ^2^ versus the mean level *E* for HIV long terminal repeat (LTR) promoter across genomic positions in Jurkat T cells can be well captured by varying the activation rate *k*_on2_ of the signaling pathway in the cross‐talk pathway model (red curve) [36, 49]. The parameters are set to be (*k*_on1_, *k*_off_, *q*_2_, *k*_b_) = (0.005, 100, 0.9999, 200). (D) Under the same parameters for fitting HIV data in (C), *J*_*i*_, with a relatively small *i*, presents a clear up‐and‐down variation with respect to the increase in *E*. As *i* increases, such up‐and‐down variation gradually reduces a monotonic increasing trend (left panel). The corresponding mRNA distribution displays a bimodal pattern. As *E* increases, the zero peak becomes higher and the distance between two peaks becomes larger (right panels). HIV, human immunodeficiency virus.

Traditional methods that measure gene transcription in cell populations have focused on how the up‐ or down‐regulation of genes engineers cell fate changes. By fitting the transcription data to infer the parameters of the telegraph model, gene expression can be upregulated by increasing the activation rate *k*_on_ for the ZRT1 gene in yeast [46], decreasing the inactivation rate *k*_off_ for over 20 E. *coli* promoters [47], and increasing the synthesis rate *k*_b_ for the serum‐induced mammalian *ctgf* gene [48]. If only one parameter of the telegraph model increases, the jumping index *J*_*i*_ strictly increases in *k*_on_ and *k*_b_ and strictly decreases in *k*_off_ [27]. This implies that the upregulation of the mean transcription level *E* is positively associated with the enhancement of cell fate change. This observation is also maintained when a single parameter varies for the three‐state model and the single selection probability *q*_2_ (or *q*_1_) and inactivation rate *k*_off_ vary for the cross‐talk pathway model; see Figure 2A,B.

To further understand why the upregulation of transcription mean *E* can enhance *J*_*i*_, we plot distributions *P*_*m*_ under different *E* values induced by changing parameters *k*_on1_ and *k*_off_ of the three‐state model or parameters *q*_2_ and *k*_off_ of the cross‐talk pathway model; see Figure 2A,B. For each pair of the distributions *P*_*m*_ under the different *E* values, we find that the two distributions intersect only once, that is, there exists an integer m* ≥ 0 such that *P*_*m*_ with higher *E* has smaller values for *m* ≤ m* while it has larger values for *m* > m*. Therefore, higher *E* gives rise to both larger $J_{i}=1-\sum_{m=0}^{i-1}P_{m}$ for 1 ≤ *i* ≤ m* + 1 and larger $J_{i}=\sum_{m=i}^{\infty }P_{m}$ for *i* > m* + 1. This explains why the increase in *E* induced by modulating some single system parameters can enhance *J*_*i*_ for all *i* ≥ 1.

Does the upregulation of transcription mean *E* always enhance *J*_*i*_? We found that the answer is negative when the upregulation is contributed by (a) changing two or more of system parameters [3, 50] and (b) changing the single activation rate *k*_on2_ of the stronger pathway in the cross‐talk pathway model [36, 43]. For circumstance (a), it is possible that the upregulation of the mean level *E* suppresses *J*_*i*_ at some *i*. For the telegraph model, when *k*_b_ and the ratio *k*_on_/*k*_off_ remain unchanged while *k*_on_ and *k*_off_ simultaneously increase, we obtain an unchanged *E* and a decrease in *J*_*i*_ for a relatively large *i* [27]. Owing to the continuous dependence of *J*_*i*_ on *k*_b_, we can slightly increase *k*_b_ to force the upregulation of *E* while still maintaining a decrease in *J*_*i*_ for large *i*.

For circumstance (b), the mean *E* increases by changing the activation rate *k*_on2_ of the stronger signaling pathway. We showed that *J*_*i*_ first increases to a unique peak but decreases thereafter at large mean levels. To illustrate this observation, we examined the regulation of cross‐talk pathways in the HIV long terminal repeat (LTR) promoter across different genomic locations [42, 43]. Figure 2C presents scattered data of noise against the mean level, where each data point corresponds to an mRNA distribution of LTR promoter at a lentiviral integration site in Jurkat T cells [49]. As the LTR promoter was developed to drive dual green fluorescent protein and ribonucleic acid smFISH reporters but not viral genes, there was no production of HIV gene products such as the *Tat* protein that forms a positive feedback network [51].

Previous studies have shown that by varying the activation rate *k*_on2_ in the cross‐talk pathway model, the data of the HIV promoter that present a down‐and‐up correlation of noise versus mean can be well captured [36]; see Figure 2C. Using the same parameter set that provided a good theoretical fit to the data, we plotted the corresponding *J*_*i*_ versus the mean level *E*. As shown in Figure 2D, *J*_*i*_, with a relatively small *i*, exhibits a significant up‐and‐down variation as *E* increases. Such an up‐and‐down variation is maintained even when *i* = 35, which is almost the largest HIV transcription level across the viral integration sites. However, when *i* increases further, the curve of *J*_*i*_ versus *E* gradually switches from nonmonotonic to monotonically increasing. This may result in the interesting observation that *J*_*i*_ with both small and large *i* may have similar values at a large mean level. To further understand why *J*_*i*_ takes similar values at both low *i* = 5 and high *i* = 35, we plot mRNA distributions under different mean levels *E* (induced by changing *k*_on2_); see Figure 2E. When *E* = 5.5 is small, the distribution is bimodal and its two peaks are very close. When *E* increases, the bimodal distribution is maintained. Specially, the zero peak is lifted up and the nonzero peak moves to the right. This enlarges the distance gradually and reduces *P*_*m*_ values between the two peaks. Therefore, under a large mean level *E* > 50, the nonzero peak of *P*_*m*_ moves to the right side of *m* = 35, and the values of *P*_*m*_, *m* ∈ [5,35] are very small, resulting in the similar values for *J*_5_ and *J*_35_.

### 2.3 Transcription noise bidirectionally influences cell fate change

A stunning finding is that transcription noise can influence cell fate changes even when the mean transcription level is not significantly up‐ or down‐regulated [20, 52, 53]. In a pioneering study linking transcriptional noise to phenotypic consequences, Blake et al. [52] measured the chances of surviving acute environmental stress in two yeast populations, one hosting the wild‐type *GAL1* promoter from *S. cerevisiae* and the other hosting a mutant *GAL1* [52]. The expression of a stress response gene in the two strains was controlled to exhibit a similar mean level but different noise levels. At higher stress levels, the higher noise strain exhibited a clear advantage in cell survival. Intriguingly, when the stress level was low, the strain with less expression noise gained a fitness benefit [52]. These observations demonstrate that transcriptional noise can influence cell fate changes bidirectionally.

To emphasize the role of transcription noise in driving cell fate changes, we focused on the transcription of the critical gene under two distinct cellular conditions that produce the same mean level by simultaneously modulating the gene activation and inactivation rates. During reactivation of HIV from latency, noise enhancers act as chromatin remodelers that stabilize both closed and open chromatin states, resulting in prolonged active and inactive HIV promoter durations with almost unchanged mean transcription levels [20]. Under acute stress, TATA box mutations with controlled ATc concentrations promote the dissociation of the activator TATA box binding protein and repressor TetR from the yeast *GAL1* promoter, which increases both the gene inactivation and activation frequencies without significantly changing the mean level [52].

In the telegraph model, to ensure an unchanged transcription mean *E* but different noise levels *CV* ^2^, we assume a constant synthesis rate *k*_b_ and reverse the first formula of Equation (4) to obtain variations in *k*_off_ and *k*_on_:

$$k_{\text{off}}=\frac{\left(k_{b}-E\right)k_{\text{on}}}{E}.$$

Under the above assumption, we took advantage of Equation (2) and showed that for any pair of different parameter sets (*k*_b_, *k*_on_, *k*_off_), there exists a unique integer *k* ≥ 2, called the *crossing digit*, such that the parameter set generating higher noise yields a larger *J*_*i*_ if *i* ≥ *k* and a smaller *J*_*i*_ if *i* < *k* [27]. This theoretical analysis confirms the noise‐induced bidirectional cell fate change. If the transcription threshold *I* of the critical gene is large more cells will change their fate in a noisier population. Conversely, if *I* is small more cells change their fate in a less noisy population.

Is a noise‐induced bidirectional cell fate change maintained when gene activation is controlled by multiple steps or cross‐talk pathways? To test this, we first fixed *k*_b_ = 30 for both the three‐state and cross‐talk pathway models. This gives the largest mean transcription level *E* = 30, as shown for over 5000 primary mouse fibroblast genes [4] and 20 *E*. *coli* promoters under different growth conditions [47]. We separately generated 15 distinct parameter sets that maintained *E* ≡ 5 and *E* ≡ 25 for both models to separately present the conditions of low and high mRNA copy numbers. Here, we randomly generated *k*_on1_, *k*_on2_ ∈ (0.1,10) for the three‐state model and *k*_on1_ ≡ 1, *k*_on2_, *k*_off_ ∈ (0.1,10) for the cross‐talk pathway model. Under these randomly generated parameters, the inactivation rate *k*_off_ ∈ (0.25, 25) of the three‐state model is computed by reversing the first formula of Equation (5) as follows:

$$k_{\text{off}}=\frac{\left(k_{b}-E\right)k_{\text{on}1}k_{\text{on}2}}{E\left(k_{\text{on}1}+k_{\text{on}2}\right)}.$$

**FIGURE 3:**
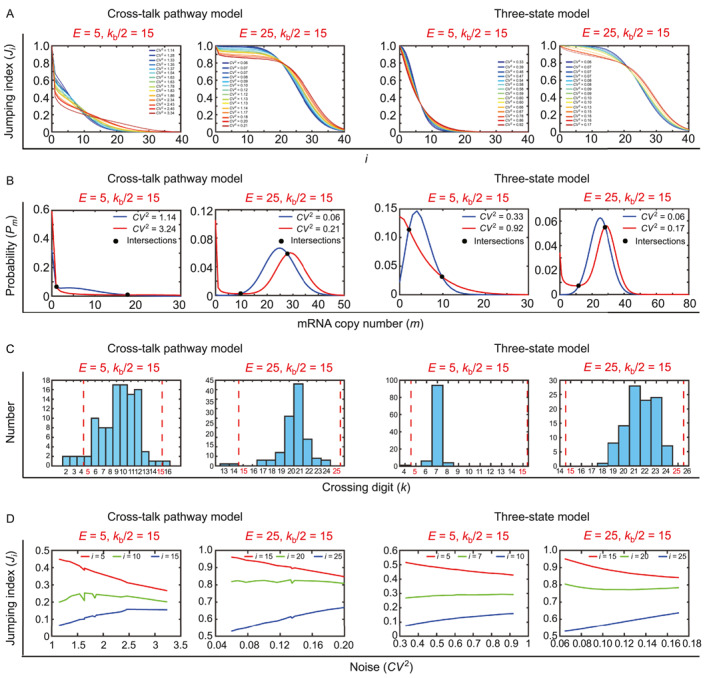
Transcription noise bidirectionally influences *J*_*i*_ for the three‐state and cross‐talk pathway models. (A) For each model, 15 parameter sets that maintain the same average level *E* = 5 or *E* = 25 but have different noise levels *CV* ^2^ were randomly generated. Each parameter set gives rise to a sequence *J*_*i*_, and each pair of *J*_*i*_ must intersect once at a unique crossing digit *k*, such that the noisier *J*_*i*_ is smaller when *i* < *k* and larger when *i* ≥ *k*. (B) For each model, a pair of mRNA distributions under the same *E* but different *CV* ^2^ intersect exactly twice. (C) 15 parameter sets of each model give rise to 15 sequences *J*_*i*_ with 105 crossing digits *k*. The values of *k* almost fall within the region between the mean level *E* and half of the synthesis rate *k*_b_ (marked by red dashed lines). (D) For each model, the increase in noise alone enhances *J*_*i*_ when $i<\min\;\{E,\;k_{b}/2\}$ and *J*_*i*_ when $i>\max\;\{E,\;k_{b}/2\}$, and it has insignificant influence on *J*_*i*_ when *i* resides between *E* and *k*_b_/2.

The selection probability *q*_2_ ∈ (0, 1) of the cross‐talk pathway model is computed by reversing the first formula of Equation (6) as follows:

$$q_{2}=\frac{\left(k_{\text{on}1}k_{b}-k_{\text{on}1}E-k_{\text{off}}E\right)k_{\text{on}2}}{k_{\text{off}}E\left(k_{\text{on}1}-k_{\text{on}2}\right)},$$

with fixed *E* = 5 or *E* = 25. As shown in Figure 3A, each parameter set of the model gives rise to a sequence *J*_*i*_,*i* = 0,1,⋯, which can be exhibited as a curve of *J*_*i*_ against *i*. For each of the three‐state and cross‐talk pathway models, each pair of sequences *J*_*i*_ shares the same mean level *E* but uses different noises *CV* ^2^. We found that each pair of *J*_*i*_ must intersect at a crossing digit *i* = *k*, and node *J*_*i*_ is smaller when *i* < *k* but larger when *i* ≥ *k*. This result is identical to that illustrated in the telegraph model [27].

To understand why the increase in transcription noise *CV* ^2^ induces the bidirectional variation of *J*_*i*_ under lower and higher *i* values as shown in Figure 3A, we plot corresponding distributions *P*_*m*_ of the three‐state and cross‐talk pathway models. Under the fixed mean level *E* and the increase of *CV* ^2^, we find that the variation in distribution patterns (bimodal, unimodal with a zero peak, and unimodal with a nonzero peak) does not follow a general principle; see Figure 3B. This suggests that the distribution pattern may not be the major reason for the bidirectional variation of *J*_*i*_. Furthermore, for each pair of *P*_*m*_ under the same *E*, we find that there exist two integers *m*_0_ ≥ 0 and *m*_1_ > *m*_0_ + 1, such that *P*_*m*_ with higher *CV* ^2^ has larger values at *m* ≤ *m*_0_ and *m* ≥ *m*_1_, while it has lower values at *m* ∈ (*m*_0_, *m*_1_). Therefore, higher *CV* ^2^ gives rise to smaller $J_{i}=1-\sum_{m=0}^{i-1}P_{m}$ for 1 < *i* < *m*_0_ + 1 and larger $J_{i}=\sum_{m=i}^{\infty }P_{m}$ for *i* ≥ *m*_1_. This indicates the bidirectional variation of *J*_*i*_ induced by the increase of the noise and that the crossing digit *i* = *k* must reside in the region (*m*_0_, *m*_1_).

It is rather challenging to estimate the exact value of *k* given a pair of parameter sets for different models; however, it is still interesting to envisage the possible region of *k* value. For each model and each fixed mean level *E*, we generated 15 sequences of *J*_*i*_ where noise *CV* ^2^ has a 3‐fold change. This provides the crossing digit *k* (intersection point of each pair of sequences *J*_*i*_) with 15!/(2!13!) = 105. We computed the percentage of different *k* values and found that the value of *k* mostly resides within the region between the mean level *E* and half of the synthesis rate *k*_b_/2; see Figure 3C. Taken together, we showed that each pair of sequence *J*_*i*_ under the same mean level *E* must intersect once at a crossing digit *k*, and *k* almost falls within the region between *E* and *k*_b_/2. These observations further imply a general rule on how transcription noise alone orchestrates *J*_*i*_, which suppresses *J*_*i*_ when $i<\min\;\{E,\;k_{b}/2\}$, enhances *J*_*i*_ when $i>\max\;\{E,\;k_{b}/2\}$, and has an insignificant influence on *J*_*i*_ when *i* lies between *E* and *k*_b_/2; see Figure 3D.

### 2.4 Noise‐induced bidirectional cell fate changes across different gene activation frameworks

**FIGURE 4:**
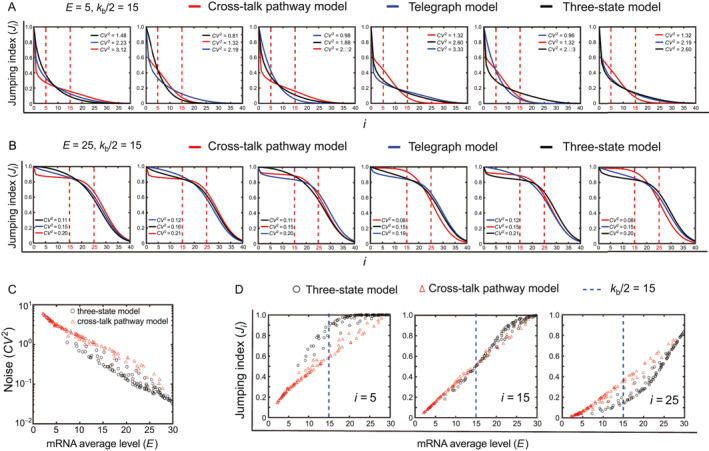
Transcription noise bidirectionally influences *J*_*i*_ across the telegraph, three‐state, and cross‐talk pathway models. (A, B) Each of the three models generates a sequence *J*_*i*_ with different noises *CV* ^2^ and exactly the same mean level *E* = 5 in (A) and *E* = 25 in (B). Each pair of *J*_*i*_ intersects at a unique crossing digit *k* and the value of *k* falls within the region between *E* and *k*_b_/2 (marked by red dashed lines). (C) 100 parameter sets are generated to obtain scattered points in *E*‐*CV* ^2^ plane for the three‐state model (black circles) and cross‐talk pathway model (red triangles). (D) Compared to the three‐state model, noisier cross‐talk pathway model generates lower *J*_*i*_ for relatively small $i<\min\;\{E,\;k_{b}/2\}$, higher *J*_*i*_ for relatively large $i>\min\;\{E,\;k_{b}/2\}$, and similar *J*_*i*_ for median *i* values.

For each of the telegraph, three‐state, and cross‐talk pathway models, we demonstrated the existence of the crossing digit *k* and its region for each pair of sequence *J*_*i*_ with the same mean level *E*. The question is whether such a rule is maintained for a pair of sequences *J*_*i*_, where a sequence *J*_*i*_ is generated by one model and the second sequence *J*_*i*_ is generated by another distinct model. To test this, we fixed *k*_b_ ≡ 30 and the mean level *E* = 5 or *E* = 25 and utilized each of the telegraph, three‐state, and cross‐talk pathway models to generate a corresponding sequence *J*_*i*_. The three sequences *J*_*i*_ generated separately by the three models have different noise levels *CV* ^2^ and each model may provide the highest, median, or lowest *CV* ^2^. There are six possibilities and we tested all possibilities under fixed mean levels *E* = 5 (Figure 4A) and *E* = 25 (Figure 4B). These observations are similar to those shown in Figure 3A,B on the intersection property for a pair of *J*_*i*_ with the same *E*.

To further test the effect of transcription noise alone on *J*_*i*_ bidirectionally across different gene activation frameworks, we fixed *k*_b_ = 30 and randomly generated 100 parameter sets for each of the three‐state and cross‐talk pathway models. We plotted the scattered points of noise *CV* ^2^ versus the mean level *E* for each model equipped with 100 randomly generated parameter sets. As shown in Figure 4C, the cross‐talk pathway model usually generates higher transcription noise than that of the three‐state model under the same mean level, confirming the previous results [33, 45]. Furthermore, we plotted the scattered points of *J*_*i*_ versus *E* for each model under 100 parameter sets (Figure 4D). This shows that the noisier cross‐talk pathway model generates a lower *J*_*i*_ for relatively small $i<\min\;\{k_{b}/2,E\}$, higher *J*_*i*_ for relatively large $i>\min\;\{k_{b}/2,E\}$, and similar *J*_*i*_ for median *i* values compared to the less noisy three‐state model.

## 3 DISCUSSIONS

In this study, we used a mathematical approach to explore how different gene activation frameworks directly affect cell fate. For each integer *i* ≥ 0, we utilize *J*_*i*_, called the jumping index, which is the probability that a cell has at least *i* mRNA molecules of a given gene. According to a recent study [27], let *I* be the statistical and measurable threshold value for the mRNA copy number of a critical gene that ascertains the entry level of Waddington’s canal toward a specific cell fate. Then, *J*_*I*_ approximates the likelihood that a cell will commit to its corresponding fate. By making use of the classical telegraph model, we showed that the upregulation of the transcription mean level can significantly enhance cell fate change whereas the enhancement of the transcription noise can navigate cell fate bidirectionally [27]. We generalize these results by considering relatively complex three‐state and cross‐talk pathway models. We found that (a) the upregulation of the critical gene may enhance the cell fate change at relatively small transcription mean levels while it may suppress the cell fate change at high transcription mean levels, and (b) increasing transcription noise alone significantly suppresses or enhances cell fate change when the threshold *I* is less or greater than both the transcription mean and half of the mRNA synthesis rate, respectively.

Our results may help illustrate how to reactivate HIV during latency, a persistent state that prevents virus eradication in most infected patients [11, 20, 51]. For HIV transcription, assuming that *I* is the threshold required for viral replication, *J*_*I*_ quantifies the HIV reactivation rate. In a seminal study [20], Dar et al. identified 85 transcriptional noise‐enhancer compounds that did not significantly alter mean levels. Noting that noise enhancers alone did not considerably alter the HIV reactivation rate [20], our observation suggests that the threshold *I* must be the median value between the basal HIV transcription mean levels and half of the synthesis rates across HIV integration sites. We provided a good fit to the HIV transcription data by modulating the activation rate of the signaling pathway, which provides an up‐and‐down variation of *J*_*I*_ versus the increasing transcription mean if *I* does not exceed the largest HIV basal transcription mean. Therefore, for HIV activators such as tumor necrosis factor (TNF), which mainly increase the gene activation frequency [50], high dosages may even suppress the HIV reactivation rate, although the mean HIV transcription level can be very high. The best strategy may be the synergism of median TNF dosages and noise enhancers to achieve better reactivation than existing reactivation drug combinations [11, 20, 27]. In contrast, for activators that modulate gene inactivation frequency, we showed that *J*_*I*_ always increases with respect to the mean level. Therefore, activators that modulate gene inactivation alone can significantly enhance the HIV reactivation rate to a large extent if their side effects are neglected [20]. However, the combination of such activators and noise enhancers should be avoided because they are predicted to generate an antagonistic effect on HIV reactivation [27].

**FIGURE 5:**
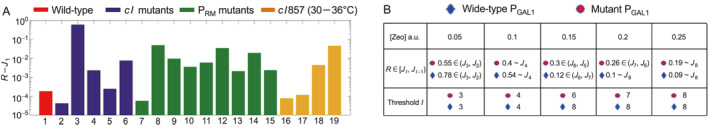
Estimation of the transcription threshold *I* of the critical gene. (A) The transcription of P_RM_‐*cl* gene plays a key role in stabilizing lambda lysogeny. For each allele of P_RM_‐*cl* [54], the lysogenic commitment probability *R* is larger than *J*_1_ (*R* − *J*_1_ > 0), suggesting *R* ∈ (*J*_1_, 1). Then the threshold is *I* ≡ 1 according to our definition. *cI*857 was measured in the temperature range 30–36°C. (B) The stress response *Sh ble* gene, controlled by wild‐type (blue diamonds) and mutant (red circles) *GAL1* promoters P_RM_‐*cl* in the two *S. cerevisiae* strains, determine the cell viability rate *R* under antibiotic Zeocin challenging [52]. For each Zeocin level ([Zeo] = 0.05, 0.1, 0.15, 0.2, 0.25 a.u.) and each strain, the threshold *I* can be estimated such that *R* ∈ (*J*_*I*_, *J*_*I*‒1_). For the two strains the thresholds *I* are almost identical under each stress condition.

Our results also illustrate how transcription noise influences cell viability under acute environmental stress. For stress response genes, if *I* is the transcription threshold for cell survival, then *J*_*I*_ approximates the cell viability rate *R* [52, 53]. The definition of *I* allows us to estimate its value using the data for *R* and *J*_*i*_ of the critical stress response gene. We first illustrated this using the lambda phage system, which has been well characterized in terms of the regulatory circuits of two key genes, *cI* and *cro*. The *cI*‐dominated lysogenic state switches to the *cro*‐dominated state (lysis) after a drastic decrease in the expression of *cI*. An interesting study by Zong et al. [54] quantified transcription of the *cI* gene using the telegraph model. For each of the 19 alleles of wild‐type P_RM_‐*cI* and its mutants in different environments, we estimated the parameters of the telegraph model (see Methods) to compute the data of *J*_*i*_ and compared *J*_*i*_ with the corresponding lysogenic rates *R*. We found that *R* always falls within the region (*J*_1_, 1) manifested by the observation that *R* − *J*_1_ > 0; see Figure 5A. Therefore, the threshold *I* ≡ 1, which suggests the occurrence of stable lysogeny once *cI* mRNA is produced, and in other words, of lysogenic switch if there is no *cI* mRNA in the cell. This is similar to a previous study [54] which concluded that the lysogenic switch to cellular lysis is determined by the probability of the cell not producing any *cI* mRNA. According to our results, increasing the transcription noise of *cI* alone significantly suppresses *J*_1_ and thus, promotes lysogenic switch to cellular lysis.

However, an increase in transcriptional noise does not always promote cell death; it may behave bidirectionally to promote cell survival. We illustrate this conclusion using classical works [52], where Blake et al. inserted the wild‐type and mutant *GAL1* promoters P_GAL1_ upstream of the stress response *Sh ble* gene in *S. cerevisiae*. The two strains were first controlled to exhibit the same mean expression but different noises and then exposed to the antibiotic Zeocin, which causes cell death. Assuming that *I* is the transcription threshold of the *Sh ble* gene, which is necessary for cell survival under zeocin stress, we estimated the system parameters of *Sh ble* gene (see the Methods section) to compute *J*_*i*_ [27]. Together with the data of viability rate *R*, we obtained the threshold *I* for *Sh ble* at each Zeocin concentration (Figure 5B) as well as the crossing digit *k* = 6 for the two sequences *J*_*i*_ corresponding to the two strains [27].

According to our results, when Zeocin concentration is less than 0.1 a.u., then *I* < *k* = 6 and the increase in transcription noise suppresses *J*_*I*_ and thus, promotes cell death. When Zeocin concentration exceeds 0.15 a.u., then *I* > *k* and increasing noise enhances *J*_*I*_ and thus, promotes cell survival. Given the above discussion, our results shed light on the circumstances under which transcription noise could be beneficial for cells under selection pressure. Stress gene promoter variants and variants with minimal noise are more likely to be selected under lower pressure to ensure a higher viability rate [55]; conversely, when the environmental pressure becomes high, the cells may favor noisier stress genes to maintain cell viability [53]. Furthermore, we confirmed that the cross‐talk pathway model usually performs noisier transcription than the three‐state model. This suggests that cells may favor ordered multiple steps or cross‐talk signaling pathways to direct stress gene activation under lower or higher environmental stress, respectively.

The current study has some limitations. First, in our three‐state or cross‐talk pathway model, we assume that there are only two sequential gene off states or two parallel signaling pathways. However, in reality, the multi‐step process of chromatin remodeling suggests multiple gene off states [33, 56]; multiple promoter binding sites or the regulation of promoter cycle suggests multiple gene on states [57, 58]; and multiple TFs binding on the promoter suggests multiple pathways to direct gene activation [36, 45]. For more general gene regulation mechanisms, we can consider non‐Markovian frameworks by introducing arbitrary duration distributions of gene on and off states [59, 60, 61, 62]. Second, in our models, we ignore one of the most common gene network motifs, the autoregulatory feedback loops whereby protein expressed from a gene activates or represses its own transcription [40, 51]. Last but not least, we only consider the steady‐state gene transcription while the cell fate change is usually tightly correlated with time due to the time‐dependent system parameters induced by cell cycles or temporal environmental changes [5, 63]. Future work is required to further test our results by adding more detailed biological mechanisms into the stochastic gene expression models.

## 4 METHODS

### 4.1 Computation of the mass function *P*_*m*_

For models discussed in the paper, *P*_*m*_ can be computed through their exact forms in Matrix laboratory. However, note that the computation of hypergeometric functions _1_ *F*_1_ and _2_ *F*_2_ may give rise to significant errors when *k*_b_ is relatively large [39, 44]. An alternative method is to utilize the finite‐state projection (FSP) algorithm to directly compute the chemical master equations of the models [5]. In this paper, we utilize exact forms and FSP algorithm to compute *P*_*m*_ when *k*_d_ ≤ 30 and *k*_d_ > 30, respectively. The accuracy of this procedure has also been verified by the stochastic simulation algorithm for 10^4^ cells (Supplementary, Figure S1).

### 4.2 Parameter estimation for P_RM_‐*cl* gene [61]

In an interesting work of *E. Coli*, Zong et al. quantified the transcription of P_RM_‐*cl* gene that plays a key role in stabilizing lambda lysogeny [54]. They revealed lysogenic rates *R* for 19 alleles of the wild‐type P_RM_‐*cl* and its mutants under different conditions, along with their transcription burst size *b* = *k*_b_/*k*_off_ and frequency *f* = 16 per cell generation using the telegraph model. Taking into account the measured degradation rate *k*_d_ = 0.25 min^−1^ for *cI* mRNA [54] and the general synthesis rate *k*_b_ = 18 min^−1^ for the promoter P_RM_ [47], we thus estimated *k*_b_/*k*_d_ = 72 and in turn obtain *k*_off_/*k*_d_ = *k*_b_/(*bk*_d_). To estimate the activation rate *k*_on_, we found that *k*_on_ = *f*/*T*_*c*_, where *T*_*c*_ is a cell generation time. For wild‐type P_RM_‐*cl*, its burst frequency takes either *f* = 16 generation^−1^ or *k*_on_ = 1.4 min^−1^ [54], indicating *T*_*c*_ = 16/1.4 min. Thus we can compute *k*_on_/*k*_d_ = 1.4*f*/(16*k*_d_) for all 19 alleles.

### 4.3 Parameter estimation for wild‐type and mutant *GAL1* promoters [52]

In a classical work of *S. cerevisiae* [52], Blake et al. constructed two strains consisting of wild‐type and mutant *GAL1* promoters P_GAL1_ upstream of a stress response gene. The two strains were first grown in galactose media and controlled to exhibit the similar expression mean *E* but different noise levels *CV* ^2^ = 1.44 for wild‐type P_GAL1_ and *CV* ^2^ = 0.36 for the mutant version. Most mRNA half‐lives in yeast range around a median of 11 min [64], so we fixed *k*_d_ = ln 2/11 ≈ 0.063 min^−1^. Promoter P_GAL1_ has transcription rate *r* ≈ 0.26 = *k*_b_× *k*_on_ (*k*_on_ + *k*_off_) < *k*_b_ in galactose‐grown condition, so we set transcription mean *E* = *r*/*k*_d_ ≈ 4 and choose *k*_d_ ≈ 2 min^−1^, the upper bound of *r* for more than 4600 genes in *S. cerevisiae* [65]. When *CV* ^2^, *E* and *k*_b_/*k*_d_ are determined, *k*_on_/*k*_d_ and *k*_off_/*k*_d_ can be derived by solving Equation (4) (*k*_b_, *k*_on_ and *k*_off_ in Equation (4) should be separately replaced by *k*_b_/*k*_d_, *k*_on_/*k*_d_ and *k*_off_/*k*_d_) and take the form

$$\frac{k_{\text{on}}}{k_{d}}=\frac{E\left(k_{b}/k_{d}+1-E\cdot {CV}^{2}-E\right)}{k_{b}/k_{d}\left(E\cdot {CV}^{2}-1\right)},\frac{k_{\text{off}}}{k_{d}}=\frac{\left(k_{\text{on}}/k_{d}\right)\left(k_{b}/k_{d}-E\right)}{E}.$$

Thus we estimated (*k*_on_/*k*_d_, *k*_off_/*k*_d_, *k*_b_/*k*_d_)≈(0.6, 4.2, 31.7) and (7.8, 54.6, 31.7) for wild‐type P_GAL1_ and its mutant version, respectively. This procedure of parameter estimation can be also found in [27].

## AUTHOR CONTRIBUTIONS

**Xinxin Chen**: Formal analysis; investigation; software. **Ying Sheng**: Data curation; resources. **Liang Chen**: Software. **Moxun Tang**: Conceptualization; funding acquisition; methodology; validation; writing—original draft. **Feng Jiao**: Conceptualization; data curation; funding acquisition; methodology; supervision; validation; writing—original draft; writing—review & editing.

## CONFLICT OF INTEREST STATEMENT

The authors Xinxin Chen, Ying Sheng, Liang Chen, Moxun Tang, and Feng Jiao declare that they have no conflicts of interest or financial conflicts to disclose.

## ETHICS STATEMENT

This article does not contain any studies with human or animal materials performed by any of the authors.

## Supporting information

Supplementary Material

## Data Availability

All data needed to evaluate the conclusions are present in the paper.

## References

[1] Larson DR . What do expression dynamics tell us about the mechanism of transcription. Curr Opin Genet Dev. 2011;21(5):591–599. 21862317 10.1016/j.gde.2011.07.010 PMC3475196

[2] Munsky B , Neuert G , Van Oudenaarden A . Using gene expression noise to understand gene regulation. Science. 2012;336(6078):183–187. 22499939 10.1126/science.1216379 PMC3358231

[3] Sanchez A , Golding I . Genetic determinants and cellular constraints in noisy gene expression. Science. 2013;342(6163):1188–1193. 24311680 10.1126/science.1242975 PMC4045091

[4] Larsson AJM , Johnsson P , Hagemann‐Jensen M , Hartmanis L , Faridani OR , Reinius B , et al. Genomic encoding of transcriptional burst kinetics. Nature. 2019;565(7738):251–254. 30602787 10.1038/s41586-018-0836-1 PMC7610481

[5] Munsky B , Fox Z , Neuert G . Integrating single‐molecule experiments and discrete stochastic models to understand heterogeneous gene transcription dynamics. Methods. 2015;85:12–21. 26079925 10.1016/j.ymeth.2015.06.009 PMC4537808

[6] Wang J , Zhang S , Lu H , Xu H . Differential regulation of alternative promoters emerges from unified kinetics of enhancer‐promoter interaction. Nat Commun. 2022;13(1):2714. 35581264 10.1038/s41467-022-30315-6 PMC9114328

[7] Chen L , Zhu C , Jiao F . A generalized moment‐based method for estimating parameters of stochastic gene transcription. Math Biosci. 2022;345:108780. 35085545 10.1016/j.mbs.2022.108780

[8] Ackermann M . A functional perspective on phenotypic heterogeneity in microorganisms. Nat Rev Microbiol. 2015;13(8):497–508. 26145732 10.1038/nrmicro3491

[9] Kaern M , Elston TC , Blake WJ , Collins JJ . Stochasticity in gene expression: from theories to phenotypes. Nat Rev Genet. 2005;6(6):451–464. 15883588 10.1038/nrg1615

[10] Mohammed H , Hernando‐Herraez I , Savino A , Scialdone A , Macaulay I , Mulas C , et al. Single‐cell landscape of transcriptional heterogeneity and cell fate decisions during mouse early gastrulation. Cell Rep. 2017;20(5):1215–1228. 28768204 10.1016/j.celrep.2017.07.009 PMC5554778

[11] Guo X , Tang T , Duan M , Zhang L , Ge H . The nonequilibrium mechanism of noise‐enhanced drug synergy in HIV latency reactivation. iScience. 2022;25(6):104358. 35620426 10.1016/j.isci.2022.104358 PMC9127169

[12] Lord ND , Norman TM , Yuan R , Bakshi S , Losick R , Paulsson J . Stochastic antagonism between two proteins governs a bacterial cell fate switch. Science. 2019;366(6461):116–120. 31604312 10.1126/science.aaw4506 PMC7526939

[13] Qiu B , Zhou T , Zhang J . Stochastic fluctuations in apoptotic threshold of tumour cells can enhance apoptosis and combat fractional killing. R Soc Open Sci. 2020;7(2):190462. 32257298 10.1098/rsos.190462 PMC7062090

[14] Lee TI , Young RA . Transcriptional regulation and its misregulation in disease. Cell. 2013;152(6):1237–1251. 23498934 10.1016/j.cell.2013.02.014 PMC3640494

[15] Moris N , Pina C , Arias AM . Transition states and cell fate decisions in epigenetic landscapes. Nat Rev Genet. 2016;17(11):693–703. 27616569 10.1038/nrg.2016.98

[16] Calo E , Quintero‐Estades JA , Danielian PS , Nedelcu S , Berman SD , Lees JA . Rb regulates fate choice and lineage commitment in vivo. Nature. 2010;466(7310):1110–1114. 20686481 10.1038/nature09264 PMC2933655

[17] Kaufman CK , Mosimann C , Fan ZP , Yang S , Thomas AJ , Ablain J , et al. A zebrafish melanoma model reveals emergence of neural crest identity during melanoma initiation. Science. 2016;351(6272):aad2197. 26823433 10.1126/science.aad2197 PMC4868069

[18] Jiao F , Sun Q , Tang M , Yu J , Zheng B . Distribution modes and their corresponding parameter regions in stochastic gene transcription. SIAM J Appl Math. 2015;75(6):2396–2420.

[19] Kalmar T , Lim C , Hayward P , Muñoz‐Descalzo S , Nichols J , Garcia‐Ojalvo J , et al. Regulated fluctuations in nanog expression mediate cell fate decisions in embryonic stem cells. PLoS Biol. 2009;7(7):e1000149. 19582141 10.1371/journal.pbio.1000149 PMC2700273

[20] Dar RD , Hosmane NN , Arkin MR , Siliciano RF , Weinberger LS . Screening for noise in gene expression identifies drug synergies. Science. 2014;344(6190):1392–1396. 24903562 10.1126/science.1250220 PMC4122234

[21] Flavahan WA , Gaskell E , Bernstein BE . Epigenetic plasticity and the hallmarks of cancer. Science. 2017;357(6348):eaal2380. 28729483 10.1126/science.aal2380 PMC5940341

[22] Wang J , Xu L , Wang E , Huang S . The potential landscape of genetic circuits imposes the arrow of time in stem cell differentiation. Biophys J. 2010;99(1):29–39. 20655830 10.1016/j.bpj.2010.03.058 PMC2895388

[23] McDonald ACH , Biechele S , Rossant J , Stanford WL . Sox17‐mediated XEN cell conversion identifies dynamic networks controlling cell‐fate decisions in embryo‐derived stem cells. Cell Rep. 2014;9(2):780–793. 25373912 10.1016/j.celrep.2014.09.026

[24] Paek AL , Liu JC , Loewer A , Forrester WC , Lahav G . Cell‐to‐cell variation in p53 dynamics leads to fractional killing. Cell. 2016;165(3):631–642. 27062928 10.1016/j.cell.2016.03.025 PMC5217463

[25] Purvis JE , Karhohs KW , Mock C , Batchelor E , Loewer A , Lahav G . p53 dynamics control cell fate. Science. 2012;336(6087):1440–1444. 22700930 10.1126/science.1218351 PMC4162876

[26] Zechner C , Nerli E , Norden C . Stochasticity and determinism in cell fate decisions. Development. 2020;147(14):dev181495. 32669276 10.1242/dev.181495

[27] Jiao F , Tang M . Quantification of transcription noise’s impact on cell fate commitment with digital resolutions. Bioinformatics. 2022;38(11):3062–3069. 35426916 10.1093/bioinformatics/btac277

[28] Raj A , Peskin CS , Tranchina D , Vargas DY , Tyagi S . Stochastic mRNA synthesis in mammalian cells. PLoS Biol. 2006;4(10):e309. 17048983 10.1371/journal.pbio.0040309 PMC1563489

[29] Nadal E , Ammerer G , Posas F . Controlling gene expression in response to stress. Nat Rev Genet. 2011;12(12):833–845. 22048664 10.1038/nrg3055

[30] Cao Z , Grima R . Analytical distributions for detailed models of stochastic gene expression in eukaryotic cells. Proc Natl Acad Sci USA. 2020;117(9):4682–4692. 32071224 10.1073/pnas.1910888117 PMC7060679

[31] Chen L , Lin G , Jiao F . Using average transcription level to understand the regulation of stochastic gene activation. R Soc Open Sci. 2022;9(2):211757. 35223065 10.1098/rsos.211757 PMC8847896

[32] Jia C , Li Y . Analytical time‐dependent distributions for gene expression models with complex promoter switching mechanisms. SIAM J Appl Math. 2023;83(4):1572–1602.

[33] Zhou T , Zhang J . Analytical results for a multistate gene model. SIAM J Appl Math. 2012;72(3):789–818.

[34] Durrett R . Probability: theory and examples. 4.1th ed. NY: Cambridge university press; 2013.

[35] Jiao F , Tang M , Yu J . Distribution profiles and their dynamic transition in stochastic gene transcription. J Differ Equations. 2013;2013254(8):3307–3328.

[36] Jiao F , Zhu C . Regulation of gene activation by competitive cross talking pathways. Biophys J. 2020;119(6):1204–1214. 32861266 10.1016/j.bpj.2020.08.011 PMC7499120

[37] Suter DM , Molina N , Gatfield D , Schneider K , Schibler U , Naef F . Mammalian genes are transcribed with widely different bursting kinetics. Science. 2011;332(6028):472–474. 21415320 10.1126/science.1198817

[38] Zimmer C , Häkkinen A , Ribeiro AS . Estimation of kinetic parameters of transcription from temporal single‐RNA measurements. Math Biosci. 2016;271:146–153. 26522167 10.1016/j.mbs.2015.10.001

[39] Chen J , Jiao F . A novel approach for calculating exact forms of mRNA distribution in single‐cell measurements. Mathematics. 2021;10(1):27.

[40] Jiao F , Li J , Liu T , Zhu Y , Che W , Bleris L , et al. What can we learn when fitting a complex gene expression model to a simple telegraph model? PLoS Comput Biol. 2024;20(5):e1012118. 38743803 10.1371/journal.pcbi.1012118 PMC11125521

[41] Tang M . The mean and noise of stochastic gene transcription. J Theor Biol. 2008;253(2):271–280. 18472111 10.1016/j.jtbi.2008.03.023

[42] Jordan A , Defechereux P , Verdin E . The site of HIV‐1 integration in the human genome determines basal transcriptional activity and response to Tat transactivation. EMBO J. 2001;20:1726–1738. 11285236 10.1093/emboj/20.7.1726 PMC145503

[43] Nixon CC , Mavigner M , Sampey GC , Brooks AD , Spagnuolo RA , Irlbeck DM , et al. Systemic HIV and SIV latency reversal via non‐canonical NF‐κB signalling in vivo. Nature. 2020;578(7793):160–165. 31969707 10.1038/s41586-020-1951-3 PMC7111210

[44] Jiao F , Sun Q , Lin G , Yu J . Distribution profiles in gene transcription activated by the cross‐talking pathway. Discrete Contin Dyn Syst B. 2019;24(6):2799–2810.

[45] Lin G , Yu J , Zhou Z , Sun Q , Jiao F . Fluctuations of mRNA distributions in multiple pathway activated transcription. Discrete Contin Dyn Syst B. 2019;24(4):1543–1568.

[46] Carey LB , van Dijk D , Sloot PM , Kaandorp JA , Segal E . Promoter sequence determines the relationship between expression level and noise. PLoS Biol. 2013;11(4):e1001528. 23565060 10.1371/journal.pbio.1001528 PMC3614515

[47] So L , Ghosh A , Zong C , Sepúlveda LA , Segev R , Golding I . General properties of transcriptional time series in *Escherichia coli* . Nat Genet. 2011;43(6):554–560. 21532574 10.1038/ng.821 PMC3102781

[48] Molina N , Suter DM , Cannavo R , Zoller B , Gotic I , Naef F . Stimulus‐induced modulation of transcriptional bursting in a single mammalian gene. Proc Natl Acad Sci USA. 2013;110(51):20563–20568. 24297917 10.1073/pnas.1312310110 PMC3870742

[49] Dey SS , Foley JE , Limsirichai P , Schaffer DV , Arkin AP . Orthogonal control of expression mean and variance by epigenetic features at different genomic loci. Mol Syst Biol. 2015;11(5):806. 25943345 10.15252/msb.20145704 PMC4461400

[50] Dar RD , Razooky BS , Singh A , Trimeloni TV , McCollum JM , Cox CD , et al. Transcriptional burst frequency and burst size are equally modulated across the human genome. Proc Natl Acad Sci USA. 2012;109(43):17454–17459. 23064634 10.1073/pnas.1213530109 PMC3491463

[51] Razooky BS , Pai A , Aull K , Rouzine IM , Weinberger LS . A hardwired HIV latency program. Cell. 2015;160(5):990–1001. 25723172 10.1016/j.cell.2015.02.009 PMC4395878

[52] Blake WJ , Balázsi G , Kohanski MA , Isaacs FJ , Murphy KF , Kuang Y , et al. Phenotypic consequences of promoter‐mediated transcriptional noise. Mol Cell. 2006;24(6):853–865. 17189188 10.1016/j.molcel.2006.11.003

[53] Liu J , Martin‐Yken H , Bigey F , Dequin S , François JM , Capp JP . Natural yeast promoter variants reveal epistasis in the generation of transcriptional‐mediated noise and its potential benefit in stressful conditions. Genome Biol Evol. 2015;7(4):969–984. 25762217 10.1093/gbe/evv047 PMC4419794

[54] Zong C , So L , Sepúlveda LA , Skinner SO , Golding I . Lysogen stability is determined by the frequency of activity bursts from the fate‐determining gene. Mol Syst Biol. 2010;6(1):440. 21119634 10.1038/msb.2010.96 PMC3010116

[55] Uphoff S , Lord ND , Okumus B , Potvin‐Trottier L , Sherratt DJ , Paulsson J . Stochastic activation of a DNA damage response causes cell‐to‐cell mutation rate variation. Science. 2016;351(6277):1094–1097. 26941321 10.1126/science.aac9786 PMC4827329

[56] Zhang J , Chen L , Zhou T . Analytical distribution and tunability of noise in a model of promoter progress. Biophys J. 2012;102(6):1247–1257. 22455907 10.1016/j.bpj.2012.02.001 PMC3309289

[57] Yang X , Wang Z , Wu Y , Zhou T , Zhang J . Kinetic characteristics of transcriptional bursting in a complex gene model with cyclic promoter structure. Math Biosci Eng. 2022;19(4):3313–3336. 35341253 10.3934/mbe.2022153

[58] Zhang J , Zhou T . Promoter‐mediated transcriptional dynamics. Biophys J. 2014;106(2):479–488. 24461023 10.1016/j.bpj.2013.12.011 PMC3907263

[59] Luo S , Zhang Z , Wang Z , Yang X , Chen X , Zhou T , et al. Inferring transcriptional bursting kinetics from single‐cell snapshot data using a generalized telegraph model. R Soc Open Sci. 2023;10(4):221057. 37035293 10.1098/rsos.221057 PMC10073913

[60] Yang X , Luo S , Zhang Z , Wang Z , Zhou T , Zhang J . Silent transcription intervals and translational bursting lead to diverse phenotypic switching. Phys Chem Chem Phys. 2022;24(43):26600–26608. 36286225 10.1039/d2cp03703c

[61] Zheng M , Qiu Z , Jiao F , Sun Q . A novel computational method for two‐state transcription model with non‐exponential ON and OFF durations. CSAIM Trans Appl Math. 2024;5(2):295–319.

[62] Zhang J , Zhou T . Stationary moments, distribution conjugation and phenotypic regions in stochastic gene transcription. Math Biosci Eng. 2019;16(5):6134–6166. 31499756 10.3934/mbe.2019307

[63] Zhang C , Jiao F . Using steady‐state formula to estimate time‐dependent parameters of stochastic gene transcription models. Biosystems. 2024;236:105128. 38280446 10.1016/j.biosystems.2024.105128

[64] Miller C , Schwalb B , Maier K , Schulz D , Dümcke S , Zacher B , et al. Dynamic transcriptome analysis measures rates of mRNA synthesis and decay in yeast. Mol Syst Biol. 2011;7(1):458. 21206491 10.1038/msb.2010.112 PMC3049410

[65] Pelechano V , Chavez S , Perez‐Ortin JE . A complete set of nascent transcription rates for yeast genes. PLoS One. 2010;5(11):e15442. 21103382 10.1371/journal.pone.0015442 PMC2982843

