# Supplementary material for: Quantifying cell fate change under different stochastic gene activation frameworks
Source: Quant Biol. 2024 Nov 21;13(1):e82. doi: 10.1002/qub2.82 (PMC12806088; doi:10.1002/qub2.82)
Supplement: Supplementary file 1 — Supplementary Material [file QUB2-13-e82-s001.pdf]

Supplementary Material —
Quantifying cell fate change under different stochastic gene activation frameworks

Xinxin Chen, Ying Sheng, Liang Chen, Moxun Tang, Feng Jiao

1 The telegraph model

In the telegraph model, the gene is assumed to randomly switch between *off* and *on* states with activation rate k_{on} and inactivation rate k_{off}, mRNA molecules are synthesized with rate k_{b} only when gene is *on* and are degraded with rate k_{d}. We suppose $k_{\text{d}} \equiv 1$ in this paper without loss of generality. We formulate the telegraph model in mathematical terms:

$$\text{gene off} \underset{k_{\text{off}}}{\overset{k_{\text{on}}}{\rightleftarrows}} \text{gene on} \xrightarrow{k_{\text{b}}} \text{mRNA} \xrightarrow{k_{\text{d}}} \emptyset. \tag{1}$$

The microstate of the gene can be represented by an ordered pair (i, m), where i is the gene state with $i = 0, 1$ corresponding to the inactive and active states, respectively, and m is the mRNA number. Let $P_{i,m}(t)$ denote the probability of having m mRNA molecules in an individual cell at time t when the gene is in state i. Then the stochastic gene expression dynamics can be described by the Markov jump process. The evolution of the Markovian dynamics is governed by the chemical master equations (CMEs):

$$\begin{cases} \dot{P}_{0,m}(t) = (m+1)P_{0,m+1}(t) + k_{\text{off}}P_{1,m}(t) - (m + k_{\text{on}})P_{0,m}(t), \\ \dot{P}_{1,m}(t) = k_{\text{b}}P_{1,m-1}(t) + (m+1)P_{1,m+1}(t) + k_{\text{off}}P_{0,m}(t) - (k_{\text{b}} + m + k_{\text{off}})P_{1,m}(t). \end{cases} \tag{2}$$

To proceed, let $P_m(t) = P_{0,m}(t) + P_{1,m}(t)$ denote the probability of having m mRNA molecules at time t. Introducing the probability generating functions

$$F_0(z,t) = \sum_{m=0}^{\infty} P_{0,m}(t)z^m, \quad F_1(z,t) = \sum_{m=0}^{\infty} P_{1,m}(t)z^m,$$

and let $F(z,t) = F_0(z,t) + F_1(z,t)$. When $t \to \infty$, the partial derivatives in (2) degrade to 0. Let

$$F_0(z) = \lim_{t\to\infty} F_0(z,t), \quad F_1(z) = \lim_{t\to\infty} F_1(z,t)$$

and $F(z) = F_1(z) + F_0(z)$. Therefore, in steady state, the CMEs given in (2) can be transformed into the following system of ordinary differential equations (ODEs):

$$\begin{cases} F_0'(z) - zF_0'(z) - k_{\text{on}}F_0(z) + k_{\text{off}}F_1(z) = 0, \\ F_1'(z) - zF_1'(z) + k_{\text{b}}zF_1(z) - (k_{\text{b}} + k_{\text{off}})F_1(z) + k_{\text{on}}F_0(z) = 0. \end{cases} \tag{3}$$

Let $z = 1$ in (3), we can obtain $k_{\text{on}}F_0(1) = k_{\text{off}}F_1(1)$. Since

$$F(1) = F_1(1) + F_0(1) = \sum_{m=0}^{\infty} (P_{0,m}(t) + P_{1,m}(t)) \cdot 1^m = \sum_{m=0}^{\infty} P_m(t) = 1,$$

we have

$$F_0(1) = \frac{k_{\text{off}}}{k_{\text{on}} + k_{\text{off}}}, \quad F_1(1) = \frac{k_{\text{on}}}{k_{\text{on}} + k_{\text{off}}}.$$

To obtain the solution of this system of ODEs, we first add the two identities in (3), we obtain $F'(z) = k_{\text{b}} F_1(z)$. Taking $F'(z) = sF_1(z)$ and $F(z) = F_1(z) + F_0(z)$ into (3), we obtain

$$k_{\text{on}} k_{\text{b}} F(z) - [k_{\text{on}} + k_{\text{off}} - (z-1)k_{\text{b}}]F'(z) + (1-z)F''(z) = 0. \tag{4}$$

Let $\bar{z} = k_{\text{b}}(z-1)$ and $F(z) = h(\bar{z})$, then we obtain $F'(z) = k_{\text{b}} h'(\bar{z})$ and $F''(z) = k_{\text{b}}^2 h''(\bar{z})$. Taking these into (4), we obtain

$$\bar{z}h''(\bar{z}) + (k_{\text{on}} + k_{\text{off}} - \bar{z})h'(\bar{z}) - k_{\text{on}} h(\bar{z}) = 0, \tag{5}$$

which is a hypergeometric differential equation. Noticing the initial values

$$h(0) = F(1) = 1, \quad h'(0) = \frac{1}{k_{\text{b}}} F'(1) = F_1(1) = \frac{k_{\text{on}}}{k_{\text{on}} + k_{\text{off}}},$$

the second-order linear ODE (5) has a unique solution given by [1]

$$h(\bar{z}) = {}_1F_1(k_{\text{on}}, k_{\text{on}} + k_{\text{off}}, \bar{z}) = \sum_{k=0}^{+\infty} \left[\frac{\Gamma(k_{\text{on}} + k)\Gamma(k_{\text{on}} + k_{\text{off}})}{\Gamma(k_{\text{on}} + k_{\text{off}} + k)\Gamma(k_{\text{on}})} \cdot \frac{\bar{z}^k}{k!} \right].$$

Then we have

$$F(z) = {}_1F_1(k_{\text{on}}, k_{\text{on}} + k_{\text{off}}, k_{\text{b}}(z-1)) = \sum_{k=0}^{+\infty} \left[\frac{\Gamma(k_{\text{on}} + k)\Gamma(k_{\text{on}} + k_{\text{off}})}{\Gamma(k_{\text{on}} + k_{\text{off}} + k)\Gamma(k_{\text{on}})} \cdot \frac{[k_{\text{b}}(z-1)]^k}{k!} \right].$$

By the reference [1], we have

$$\frac{\mathrm{d}^m}{\mathrm{d}z^m} {}_1F_1(k_{\text{on}}, k_{\text{on}} + k_{\text{off}}, k_{\text{b}}(z-1)) = \frac{\Gamma(k_{\text{on}} + m)\Gamma(k_{\text{on}} + k_{\text{off}})k_{\text{b}}^m}{\Gamma(k_{\text{on}} + k_{\text{off}} + m)\Gamma(k_{\text{on}})} \cdot {}_1F_1(k_{\text{on}} + m, k_{\text{on}} + k_{\text{off}} + m, k_{\text{b}}(z-1)).$$

Then we obtain

$$\begin{aligned}
P_m &= \frac{1}{m!} \frac{\mathrm{d}^m F(z)}{\mathrm{d}^m z}\Big|_{z=0} \\
&= \frac{\Gamma(k_{\text{on}} + m)\Gamma(k_{\text{on}} + k_{\text{off}})}{\Gamma(k_{\text{on}} + k_{\text{off}} + m)\Gamma(k_{\text{on}})} \cdot \frac{k_{\text{b}}^m}{m!} \cdot {}_1F_1(k_{\text{on}} + m, k_{\text{on}} + k_{\text{off}} + m, k_{\text{b}}(z-1)) \\
&= \frac{\Gamma(k_{\text{on}} + m)\Gamma(k_{\text{on}} + k_{\text{off}})}{\Gamma(k_{\text{on}} + k_{\text{off}} + m)\Gamma(k_{\text{on}})} \cdot \frac{k_{\text{b}}^m}{m!} \cdot \sum_{k=0}^{+\infty} \left[\frac{\Gamma(k_{\text{on}} + m + k)\Gamma(k_{\text{on}} + k_{\text{off}} + m)}{\Gamma(k_{\text{on}} + k_{\text{off}} + m + k)\Gamma(k_{\text{on}} + m)} \cdot \frac{(-k_{\text{b}})^k}{k!} \right].
\end{aligned}$$

2 The three-state model

The three-state model consists of random rotation between two sequential gene *off* states and one *on* state with activation rates $k_{\text{on}1}, k_{\text{on}2}$ and inactivation rate k_{off}, mRNA molecules are synthesized with rate k_{b} only when gene is *on* and are degraded with rate k_{d}. Let $P_{i,m}(t)$ denote the probability of having m copies of mRNA at time t when the promoter is in state i. Here $i = 1$, $i = 2$ and $i = 3$ correspond to the first *off* state, the second *off* state and *on* state of the promoter, respectively. Now we introduce the CMEs for mRNA (suppose that the change in the mRNA number over time is Markovian) and use m to denote the number of mRNA, then the CMEs read as

$$\begin{cases}
\dot{P}_{1,m}(t) = k_{\text{off}} P_{3,m}(t) + (m+1)k_{\text{d}} P_{1,m+1}(t) - (mk_{\text{d}} + k_{\text{on}1})P_{1,m}(t), \\
\dot{P}_{2,m}(t) = k_{\text{on}1} P_{1,m}(t) + (m+1)k_{\text{d}} P_{2,m+1}(t) - (mk_{\text{d}} + k_{\text{on}2})P_{2,m}(t), \\
\dot{P}_{3,m}(t) = k_{\text{on}2} P_{2,m}(t) + k_{\text{b}} P_{3,m-1}(t) + (m+1)k_{\text{d}} P_{3,m+1}(t) - (k_{\text{b}} + mk_{\text{d}} + k_{\text{off}})P_{3,m}(t).
\end{cases} \tag{6}$$

We suppose $k_\text{d} \equiv 1$ for convenience. Here we study its steady-state solution. We define generating functions:

$$F_i(z,t) = \sum_{n=0}^{\infty} P_{i,m}(t) z^n, \quad i = 1,2,3.$$

Moreover, let $P_m(t) = P_{1,m}(t) + P_{2,m}(t) + P_{3,m}(t)$ denote the probability of having m copies of mRNA at time t and let $F(z,t) = F_1(z,t) + F_2(z,t) + F_3(z,t)$ denote its generating function. At the steady state, $F_1(z,t), F_2(z,t)$ and $F_3(z,t)$ are independent of time t and the CMEs (6) can be transformed into the following system of ODEs:

$$\begin{cases} (1-z)F_1'(z) - k_{\text{on}1}F_1(z) + k_\text{off}F_3(z) = 0, \\ (1-z)F_2'(z) - k_{\text{on}2}F_2(z) + k_{\text{on}1}F_1(z) = 0, \\ (1-z)F_3'(z) + k_\text{b}(z-1)F_3(z) - k_\text{off}F_3(z) + k_{\text{on}2}F_2(z) = 0. \end{cases} \tag{7}$$

Let $z = 1$ in (7), we can obtain $k_{\text{on}1}F_1(1) = k_{\text{on}2}F_2(1) = k_\text{off}F_3(1)$. Since

$$F(1) = F_1(1) + F_2(1) + F_3(1) = \sum_{m=0}^{\infty} (P_{1,m}(t) + P_{2,m}(t) + P_{3,m}(t)) \cdot 1^m = \sum_{m=0}^{\infty} P_m(t) = 1,$$

we obtain

$$F_1(1) = \frac{k_{\text{on}2}k_\text{off}}{\alpha\beta}, \quad F_2(1) = \frac{k_{\text{on}1}k_\text{off}}{\alpha\beta}, \quad F_3(1) = \frac{k_{\text{on}1}k_{\text{on}2}}{\alpha\beta},$$

where we set

$$\alpha\beta = k_{\text{on}1}k_{\text{on}2} + k_{\text{on}1}k_\text{off} + k_{\text{on}2}k_\text{off}, \quad \alpha + \beta = k_{\text{on}1} + k_{\text{on}2} + k_\text{off}.$$

Adding the three identities in (7), we obtain $F'(z) = k_\text{b}F_3(z)$. Taking the derivative of (7) yields

$$\begin{cases} (1-z)F_1''(z) - (1+k_{\text{on}1})F_1'(z) + k_\text{off}F_3'(z) = 0, \\ (1-z)F_2''(z) - (1+k_{\text{on}2})F_2'(z) + k_\text{off}F_3'(z) = 0, \\ k_{\text{on}2}F_2'(z) = (k_\text{off}+1)F_3'(z) + (z-1)F_3''(z) - (z-1)k_\text{b}F_3'(z) - k_\text{b}F_3(z). \end{cases} \tag{8}$$

Let $z = 1$ in (12), we can obtain $(1+k_{\text{on}1})F_1'(1) = (1+k_{\text{on}2})F_2'(1) = k_\text{off}F_3'(1)$. Since

$$F_1'(1) + F_2'(1) + F_3'(1) = F'(1) = k_\text{b}F_3(1) = \frac{k_\text{b}k_{\text{on}1}k_{\text{on}2}}{\alpha\beta},$$

we obtain

$$F_1'(1) = \frac{k_\text{b}k_{\text{on}1}k_{\text{on}2}k_\text{off}(1+k_{\text{on}2})}{\alpha\beta[(\alpha+1)(\beta+1)+k_\text{off}]}, \; F_2'(1) = \frac{k_\text{b}k_{\text{on}1}k_{\text{on}2}k_\text{off}(1+k_{\text{on}1})}{\alpha\beta[(\alpha+1)(\beta+1)+k_\text{off}]}, \; F_3'(1) = \frac{k_\text{b}k_{\text{on}1}k_{\text{on}2}(1+k_{\text{on}1})(1+k_{\text{on}2})}{\alpha\beta[(\alpha+1)(\beta+1)+k_\text{off}]}.$$

Inserting $F(z) = F_1(z) + F_2(z) + F_3(z)$ into the third equation of (7), and then multiplying the equation to $k_{\text{on}1}$, we have

$$k_{\text{on}1}k_{\text{on}2}F(z) - k_{\text{on}2}k_{\text{on}1}F_1(z) + (z-1)k_\text{b}k_{\text{on}1}F_3(z) - k_{\text{on}1}(k_\text{off}+k_{\text{on}2})F_3''(z) + k_{\text{on}1}(1-z)F_3'(z) = 0.$$

To proceed, taking the third equation of (12), the second and the third equation of (7) and $F'(z) = k_\text{b}F_3(z)$ into the above equation, we obtain

$$\begin{aligned} &(z-1)^2F'''(z) + (z-1)(k_{\text{on}1} + k_{\text{on}2} + k_\text{off} + 1 - (z-1)k_\text{b})F''(z) \\ &+ (k_{\text{on}1}k_{\text{on}2} + k_{\text{on}1}k_\text{off} + k_{\text{on}2}k_\text{off} - (k_{\text{on}1} + k_{\text{on}2} + 1)(z-1)k_\text{b})F'(z) - k_{\text{on}1}k_{\text{on}2}k_\text{b}F(z) = 0. \end{aligned}$$

Let $\bar{z} = k_{\rm b}(z-1)$ and $F(z) = h(\bar{z})$, then the above equation can be rewritten as

$$\begin{aligned}&\bar{z}^2 h'''(\bar{z}) + \bar{z}(1 - \bar{z} + k_{\rm on1} + k_{\rm on2} + k_{\rm off})h''(\bar{z})\\ &+[k_{\rm on1}k_{\rm on2} + k_{\rm on1}k_{\rm off} + k_{\rm on2}k_{\rm off} - (k_{\rm on1} + k_{\rm on2} + 1)\bar{z}]h'(\bar{z}) - k_{\rm on1}k_{\rm on2}h(\bar{z}) = 0. \end{aligned}\tag{9}$$

Noticing the initial values

$$\begin{cases} h(0) = F(1) = 1, \\ h'(0) = \dfrac{1}{k_b}F'(1) = F_3(1) = \dfrac{k_{\rm on1}k_{\rm on2}}{\alpha\beta}, \\ h''(0) = \dfrac{1}{k_b^2}F''(1) = \dfrac{1}{k_b}F_3'(1) = \dfrac{k_{\rm on1}k_{\rm on2}(1+k_{\rm on1})(1+k_{\rm on2})}{\alpha\beta[(\alpha+1)(\beta+1)+k_{\rm off}]}, \end{cases}$$

then the third-order linear ODE (9) has a unique solution $h(\bar{z}) = {}_2F_2(k_{\rm on1}, k_{\rm on2}, \alpha, \beta, \bar{z})$. Then we obtain

$$F(z) = {}_2F_2(k_{\rm on1}, k_{\rm on2}, \alpha, \beta, k_{\rm b}(z-1)) = \sum_{k=0}^{+\infty}\left[\frac{\Gamma(k_{\rm on1}+k)\Gamma(k_{\rm on2}+k)\Gamma(\alpha)\Gamma(\beta)}{\Gamma(\alpha+k)\Gamma(\beta+k)\Gamma(k_{\rm on1})\Gamma(k_{\rm on2})}\cdot\frac{[k_{\rm b}(z-1)]^k}{k!}\right].$$

By the reference [1], we have

$$\begin{aligned}\frac{{\rm d}^m}{{\rm d}z^m}{}_2F_2(k_{\rm on1}, k_{\rm on2}, \alpha, \beta, k_{\rm b}(z-1)) =&\frac{\Gamma(k_{\rm on1}+m)\Gamma(k_{\rm on2}+m)\Gamma(\alpha)\Gamma(\beta)}{\Gamma(\alpha+m)\Gamma(\beta+m)\Gamma(k_{\rm on1})\Gamma(k_{\rm on2})}\\ &\cdot {}_2F_2(k_{\rm on1}+m, k_{\rm on2}+m, \alpha+m, \beta+m, k_{\rm b}(z-1)).\end{aligned}$$

Then we obtain

$$\begin{aligned}P_m &= \frac{1}{m!}\frac{{\rm d}^m F(z)}{{\rm d}^m z}\Big|_{z=0}\\ &= \frac{\Gamma(k_{\rm on1}+m)\Gamma(k_{\rm on2}+m)\Gamma(\alpha)\Gamma(\beta)}{\Gamma(\alpha+m)\Gamma(\beta+m)\Gamma(k_{\rm on1})\Gamma(k_{\rm on2})}\cdot\frac{k_{\rm b}^m}{m!}\cdot {}_2F_2(k_{\rm on1}+m, k_{\rm on2}+m, \alpha+m, \beta+m, k_{\rm b}(z-1))\\ &= \frac{\Gamma(k_{\rm on1}+m)\Gamma(k_{\rm on2}+m)\Gamma(\alpha)\Gamma(\beta)}{\Gamma(\alpha+m)\Gamma(\beta+m)\Gamma(k_{\rm on1})\Gamma(k_{\rm on2})}\cdot\frac{k_{\rm b}^m}{m!}\\ &\cdot\sum_{k=0}^{+\infty}\left[\frac{\Gamma(k_{\rm on1}+m+k)\Gamma(k_{\rm on2}+m+k)\Gamma(\alpha+m)\Gamma(\beta+m)}{\Gamma(\alpha+m+k)\Gamma(\beta+m+k)\Gamma(k_{\rm on1}+m)\Gamma(k_{\rm on2}+m)}\cdot\frac{(-k_{\rm b})^k}{k!}\right].\end{aligned}$$

3 The cross-talk pathway model

For the cross-talk pathway model, the gene activation is induced by two parallel pathways from gene *off* state to gene *on* state. The residence times in two pathways are independent and exponentially distributed, with the induction strengths $k_{\rm on1}$ for the weak basal pathway and $k_{\rm on2}$ for the strong signaling pathway, satisfying $0 < k_{\rm on1} < k_{\rm on2} < +\infty$. The strong signaling pathway and the weak basal pathway are selected with probabilities q_1 and q_2, where $0 < q_1, q_2 < 1$ and $q_1 + q_2 = 1$. The gene randomly switch from *off* state to *on* state with inactivation rate $k_{\rm off}$, mRNA molecules are synthesized with rate $k_{\rm b}$ only when gene is *on* and are degraded with rate $k_{\rm d}$. We also set $k_{\rm d} \equiv 1$ for convenience. Let *off1* denote the gene off state in which the transition from *off* state to *on* state is induced by the weak pathway, and *off2* otherwise. Then we let $P_{i,m}(t)$ denote the probability of having m copies of mRNA at time t when the promoter is in state i. Here $i = 1$, $i = 2$ and $i = 3$ correspond to *off1* state, *off2* state and *on* state of the promoter, respectively. Then the total probability mass function $P_m(t) = P_{1,m}(t) + P_{2,m}(t) + P_{3,m}(t)$ denote the probability of having m copies

of mRNA at time t. Then the evolution of the cross-talk pathway model is governed by the CMEs:

$$\begin{cases} \dot{P}_{1,m}(t) = q_1 k_{\text{off}} P_{3,m}(t) + (m+1)P_{1,m+1}(t) - (m + k_{\text{on}1})P_{1,m}(t), \\ \dot{P}_{2,m}(t) = q_2 k_{\text{off}} P_{3,m}(t) + (m+1)P_{2,m+1}(t) - (m + k_{\text{on}2})P_{2,m}(t), \\ \dot{P}_{3,m}(t) = k_{\text{on}1} P_{1,m}(t) + k_{\text{on}2} P_{2,m}(t) + k_{\text{b}} P_{3,m-1}(t) + (m+1)P_{3,m+1}(t) - (k_{\text{b}} + m + k_{\text{off}})P_{3,m}(t). \end{cases} \tag{10}$$

We introduce the probability generating functions

$$F_i(z,t) = \sum_{m=0}^{\infty} P_{i,m}(t) z^m, \quad i = 1,2,3,$$

and let $F(z,t) = F_1(z,t) + F_2(z,t) + F_3(z,t)$. Then at the steady state, we can transform (10) into the following system of ODEs:

$$\begin{cases} (1-z)F_1'(z) - k_{\text{on}1} F_1(z) + q_1 k_{\text{off}} F_3(z) = 0, \\ (1-z)F_2'(z) - k_{\text{on}2} F_2(z) + q_2 k_{\text{off}} F_3(z) = 0, \\ (1-z)F_3'(z) + k_{\text{b}}(z-1)F_3(z) - k_{\text{off}} F_3(z) + k_{\text{on}1} F_1(z) + k_{\text{on}2} F_2(z) = 0. \end{cases} \tag{11}$$

Let $z = 1$ in (11), we can obtain $k_{\text{on}1} F_1(1) = q_1 k_{\text{off}} F_3(1)$ and $k_{\text{on}2} F_2(1) = q_2 k_{\text{off}} F_3(1)$, where $q_1 + q_2 = 1$. Since

$$F(1) = F_1(1) + F_2(1) + F_3(1) = \sum_{m=0}^{\infty} (P_{1,m}(t) + P_{2,m}(t) + P_{3,m}(t)) \cdot 1^m = \sum_{m=0}^{\infty} P_m(t) = 1,$$

we obtain

$$F_1(1) = \frac{q_1 k_{\text{on}2} k_{\text{off}}}{\bar{\alpha}\bar{\beta}}, \quad F_2(1) = \frac{q_2 k_{\text{on}1} k_{\text{off}}}{\bar{\alpha}\bar{\beta}}, \quad F_3(1) = \frac{k_{\text{on}1} k_{\text{on}2}}{\bar{\alpha}\bar{\beta}},$$

where we set

$$\bar{\alpha}\bar{\beta} = k_{\text{on}1} k_{\text{on}2} + q_2 k_{\text{on}1} k_{\text{off}} + q_1 k_{\text{on}2} k_{\text{off}}, \quad \bar{\alpha} + \bar{\beta} = k_{\text{on}1} + k_{\text{on}2} + k_{\text{off}}.$$

Taking the derivative of (11) yields

$$\begin{cases} (1-z)F_1''(z) - (1 + k_{\text{on}1})F_1'(z) + q_1 k_{\text{off}} F_3'(z) = 0, \\ (1-z)F_2''(z) - (1 + k_{\text{on}2})F_2'(z) + q_2 k_{\text{off}} F_3'(z) = 0, \\ (1-z)F_3''(z) - (1 + k_{\text{off}})F_3'(z) + k_{\text{b}}(z-1)F_3'(z) + k_{\text{b}} F_3(z) + k_{\text{on}1} F_1'(z) + k_{\text{on}2} F_2'(z) = 0. \end{cases} \tag{12}$$

Let $z = 1$ in (12), we can obtain $(1 + k_{\text{on}1})F_1'(1) = q_1 k_{\text{off}} F_3'(1)$ and $(1 + k_{\text{on}2})F_2'(1) = q_2 k_{\text{off}} F_3'(1)$. Since

$$F_1'(1) + F_2'(1) + F_3'(1) = F'(1) = k_{\text{b}} F_3(1) = \frac{k_{\text{b}} k_{\text{on}1} k_{\text{on}2}}{\bar{\alpha}\bar{\beta}},$$

we obtain

$$F_1'(1) = \frac{q_1 k_{\text{b}} k_{\text{on}1} k_{\text{on}2} k_{\text{off}} (1 + k_{\text{on}2})}{\bar{\alpha}\bar{\beta}(\bar{\alpha}+1)(\bar{\beta}+1)}, \ F_2'(1) = \frac{q_2 k_{\text{b}} k_{\text{on}1} k_{\text{on}2} k_{\text{off}} (1 + k_{\text{on}1})}{\bar{\alpha}\bar{\beta}(\bar{\alpha}+1)(\bar{\beta}+1)}, \ F_3'(1) = \frac{k_{\text{b}} k_{\text{on}1} k_{\text{on}2} (1 + k_{\text{on}1})(1 + k_{\text{on}2})}{\bar{\alpha}\bar{\beta}(\bar{\alpha}+1)(\bar{\beta}+1)}.$$

Adding the three identities in (11), we obtain $F'(z) = k_{\text{b}} F_3(z)$. To obtain the solution of (11), we first taking $F(z) = F_1(z) + F_2(z) + F_3(z)$ into the third eqution of (11), which gives

$$(k_{\text{on}2} - k_{\text{on}1})F_2(z) = -(z-1)k_{\text{b}} F_3(z) + (z-1)F_3'(z) + (k_{\text{off}} + k_{\text{on}1})F_3(z) - k_{\text{on}1} F(z).$$

Taking the above equation into the second equation of (11) and using $F'(z) = k_\mathrm{b}F_3(z)$, we have

$$\begin{aligned}
&(z-1)^2F'''(z) + (z-1)(k_{\mathrm{on}1} + k_{\mathrm{on}2} + k_\mathrm{off} + 1 - (z-1)k_\mathrm{b})F''(z) \\
&+ (k_{\mathrm{on}1}k_{\mathrm{on}2} + q_2k_{\mathrm{on}1}k_\mathrm{off} + q_1k_{\mathrm{on}2}k_\mathrm{off} - (k_{\mathrm{on}1} + k_{\mathrm{on}2} + 1)(z-1)k_\mathrm{b})F'(z) - k_{\mathrm{on}1}k_{\mathrm{on}2}k_\mathrm{b}F(z) = 0.
\end{aligned}$$

Let $\bar{z} = k_\mathrm{b}(z-1)$ and $F(z) = h(\bar{z})$, then the above equation can be rewritten as

$$\begin{aligned}
&\bar{z}^2h'''(\bar{z}) + \bar{z}(1 - \bar{z} + k_{\mathrm{on}1} + k_{\mathrm{on}2} + k_\mathrm{off})h''(\bar{z}) \\
&+[k_{\mathrm{on}1}k_{\mathrm{on}2} + q_2k_{\mathrm{on}1}k_\mathrm{off} + q_1k_{\mathrm{on}2}k_\mathrm{off} - (k_{\mathrm{on}1} + k_{\mathrm{on}2} + 1)\bar{z}]h'(\bar{z}) - k_{\mathrm{on}1}k_{\mathrm{on}2}h(\bar{z}) = 0.
\end{aligned} \tag{13}$$

Noticing the initial values

$$\begin{cases}
h(0) = F(1) = 1, \\
h'(0) = \dfrac{1}{k_b}F'(1) = F_3(1) = \dfrac{k_{\mathrm{on}1}k_{\mathrm{on}2}}{\bar{\alpha}\bar{\beta}}, \\
h''(0) = \dfrac{1}{k_b^2}F''(1) = \dfrac{1}{k_b}F_3'(1) = \dfrac{k_{\mathrm{on}1}k_{\mathrm{on}2}(1+k_{\mathrm{on}1})(1+k_{\mathrm{on}2})}{\bar{\alpha}\bar{\beta}(\bar{\alpha}+1)(\bar{\beta}+1)},
\end{cases}$$

then the third-order linear ODE (13) has a unique solution $h(\bar{z}) = {}_2F_2(k_{\mathrm{on}1}, k_{\mathrm{on}2}, \bar{\alpha}, \bar{\beta}, \bar{z})$ [1], and we can obtain

$$F(z) = {}_2F_2(k_{\mathrm{on}1}, k_{\mathrm{on}2}, \bar{\alpha}, \bar{\beta}, k_\mathrm{b}(z-1)) = \sum_{k=0}^{+\infty}\left[\frac{\Gamma(k_{\mathrm{on}1}+k)\Gamma(k_{\mathrm{on}2}+k)\Gamma(\bar{\alpha})\Gamma(\bar{\beta})}{\Gamma(\bar{\alpha}+k)\Gamma(\bar{\beta}+k)\Gamma(k_{\mathrm{on}1})\Gamma(k_{\mathrm{on}2})}\cdot\frac{[k_\mathrm{b}(z-1)]^k}{k!}\right].$$

Similar to the three-state model, we can obtain

$$\begin{aligned}
P_m &= \frac{\Gamma(k_{\mathrm{on}1}+m)\Gamma(k_{\mathrm{on}2}+m)\Gamma(\bar{\alpha})\Gamma(\bar{\beta})}{\Gamma(\bar{\alpha}+m)\Gamma(\bar{\beta}+m)\Gamma(k_{\mathrm{on}1})\Gamma(k_{\mathrm{on}2})}\cdot\frac{k_\mathrm{b}^m}{m!}\cdot{}_2F_2(k_{\mathrm{on}1}+m, k_{\mathrm{on}2}+m, \bar{\alpha}+m, \bar{\beta}+m, k_\mathrm{b}(z-1)) \\
&= \frac{\Gamma(k_{\mathrm{on}1}+m)\Gamma(k_{\mathrm{on}2}+m)\Gamma(\bar{\alpha})\Gamma(\bar{\beta})}{\Gamma(\bar{\alpha}+m)\Gamma(\bar{\beta}+m)\Gamma(k_{\mathrm{on}1})\Gamma(k_{\mathrm{on}2})}\cdot\frac{k_\mathrm{b}^m}{m!} \\
&\cdot\sum_{k=0}^{+\infty}\left[\frac{\Gamma(k_{\mathrm{on}1}+m+k)\Gamma(k_{\mathrm{on}2}+m+k)\Gamma(\bar{\alpha}+m)\Gamma(\bar{\beta}+m)}{\Gamma(\bar{\alpha}+m+k)\Gamma(\bar{\beta}+m+k)\Gamma(k_{\mathrm{on}1}+m)\Gamma(k_{\mathrm{on}2}+m)}\cdot\frac{(-k_\mathrm{b})^k}{k!}\right].
\end{aligned}$$

References

[1] Olver, F. W. J. et al. NIST Digital Library of Mathematical Functions. http://dlmf.nist.gov/, Release 1.0.17 of 2017-12-22 (2017).

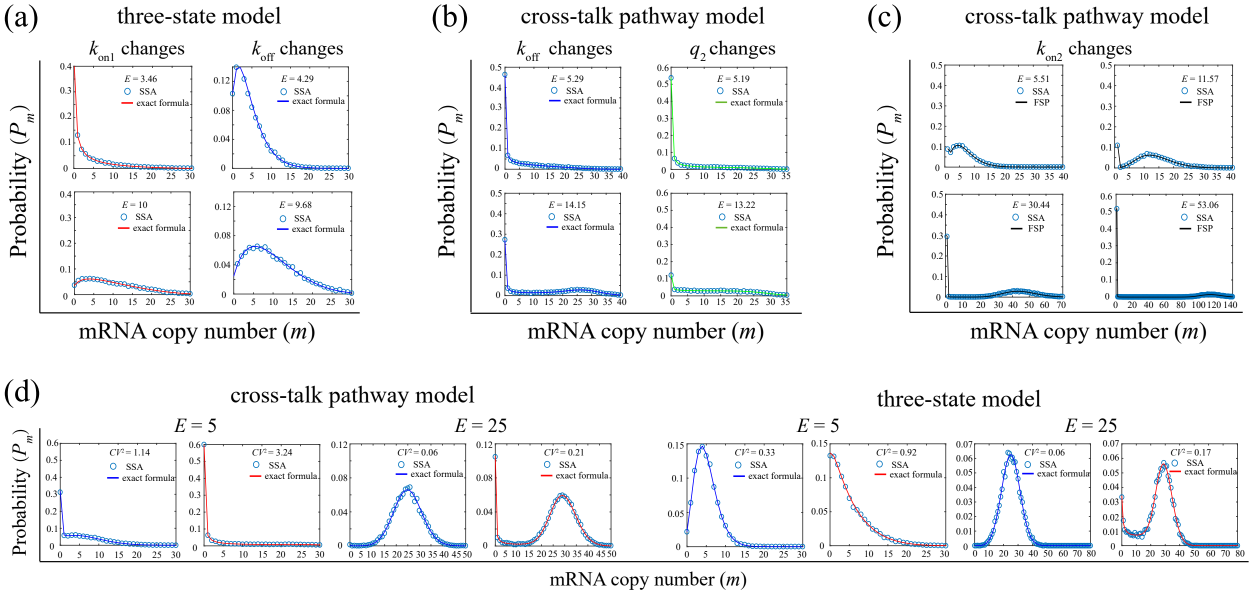

Figure 1: Test the accuracy of the computation of the distribution P_m. We utilize exact forms and finite-state projection (FSP) algorithm to compute the distribution P_m when $k_b \leq 30$ and $k_b > 30$, respectively (piecewise continuous lines). We also utilize stochastic simulation algorithm (SSA) for 10^4 cell samples to compute P_m (hollow circles), which matches accurately with our theoretical computation of P_m. (a) P_m shown in Fig. 2(a) of the main text. (b) P_m shown in Fig. 2(b) of the main text. (c) P_m shown in Fig. 2(d) of the main text. (e) P_m shown in Fig. 3(b) of the main text.
